# Infant feeding in the context of HIV: a qualitative study of health care workers’ knowledge of recommended infant feeding options in Papua New Guinea

**DOI:** 10.1186/1746-4358-8-6

**Published:** 2013-06-07

**Authors:** Lisa M Vallely, Angela Kelly, Martha Kupul, Ruthy Neo, Voletta Fiya, John M Kaldor, Glen DL Mola, Heather Worth

**Affiliations:** 1 Sexual & Reproductive Health Unit, Papua New Guinea Institute of Medical Research, Goroka, Eastern Highlands Province, Papua New Guinea; 2 International HIV Research Group, School of Public Health and Community Medicine, University of New South Wales, Sydney, Australia; 3 Kirby Institute, University of New South Wales, Sydney, Australia; 4 School of Medicine and Health Sciences, University of Papua New Guinea, Port Moresby, Papua New Guinea

**Keywords:** Prevention of mother to child transmission, Infant feeding practices, Exclusive breastfeeding, Health care worker knowledge

## Abstract

**Background:**

Interventions to prevent mother to child transmission of human immunodeficiency virus (HIV) during childbirth and breastfeeding can reduce HIV infections in infants to less than 5% in low and middle income countries. The World Health Organization (WHO) recommends all mothers, regardless of their HIV status, practice exclusive breastfeeding for the first six months of an infant’s life. In line with these recommendations and to protect, promote and support breastfeeding, in 2009 the PNG National Department of Health revised their National HIV infant feeding guidelines, reinforcing the WHO recommendation of exclusive breastfeeding for the first six months followed by the introduction of other food and fluids, while continuing breastfeeding.

The overall aim of this paper is to explore health care workers’ knowledge regarding infant feeding options in PNG, specifically as they relate to HIV exposed infants.

**Methods:**

As part of a study investigating women’s and men’s experiences of prevention of mother to child transmission (PMTCT) services in two sites in PNG, 28 key informant interviews were undertaken. This paper addresses one theme that emerged from thematic data analysis: Health care workers’ knowledge regarding infant feeding options, specifically how this knowledge reflects the Papua New Guinea National HIV Care and Treatment Guidelines on HIV and infant feeding (2009).

**Results:**

Most informants mentioned exclusive breastfeeding, the majority of whom reflected the most up-to-date National Guidelines of exclusive breastfeeding for six months. The importance of breastfeeding continuing beyond this time, along with the introduction of food and fluids was less well understood. The most senior people involved in PMTCT were the informants who most accurately reflected the national guidelines of continuing breastfeeding after six months.

**Conclusion:**

Providing advice on optimal infant feeding in resource poor settings is problematic, especially in relation to HIV transmission. Findings from our study reflect those found elsewhere in identifying that key health care workers are not aware of up-to-date information relating to infant feeding, especially within the context of HIV. Greater emphasis needs to be placed on ensuring the most recent feeding guidelines are disseminated and implemented in clinical practice in PNG.

## Background

Vertical transmission is the leading source of human immunodeficiency virus (HIV) in children under the age of 15 years [1]. Without intervention 30-45% of infants born to HIV-positive mothers in developing countries will become infected during pregnancy, childbirth and breastfeeding [2]. Interventions to prevent mother-to-child transmission during childbirth and breastfeeding have been shown to reduce infections in infants to less than 5% in low and middle income countries [3-5].

The superiority of breastfeeding over artificial feeding is well documented and exclusive breastfeeding remains one of the most valuable interventions for improving child survival, especially in resource poor settings [6]. The transmission of HIV through breastfeeding was first identified in 1985 [7] and since then the issue of breastfeeding within the context of HIV and prevention of mother to child transmission (PMTCT) has continued to be at the centre of much debate and policy. The World Health Organization (WHO) recommends all mothers, regardless of their HIV status practice exclusive breastfeeding for the first six months of an infant’s life.

In the absence antiretroviral therapy (ART) the risk of mother-to-child transmission of HIV through breastfeeding is between 20-45% [8]. However, with the use of ART by the mother this risk can be reduced to less than 5%, even among infants who are breastfed [9-11], because ART reduces the HIV viral load in the mother’s milk [12]. While breastfeeding is associated with risk of transmission of HIV [13], exclusive breastfeeding for the first six months is associated with a lower risk of HIV transmission when compared to mixed feeding, even without ART [3,4]. The risk of infants acquiring HIV through breastfeeding, therefore needs to be weighed against the increased risk of death from causes other than HIV, in particular malnutrition and serious illnesses such as diarrhoea, among non-breastfed infants [14].

In order to continue encouraging and promoting breastfeeding while at the same time reducing the risk of HIV transmission, global infant feeding guidelines relating to HIV-exposed infants have been developed and subsequently amended as knowledge and understanding of HIV and its biomedical management has advanced [15-20]. These guidelines are particularly relevant for HIV-positive mothers in low income countries [21]. While clearly favouring breastfeeding, the guidelines note that in some situations replacement feeding may be more appropriate, providing it is “acceptable, feasible, affordable, sustainable and safe”. If these provisions cannot be met, exclusive breastfeeding in the first few months of life (currently the first six months) is recommended [17,19]. While earlier WHO guidelines (2001) recommended abrupt cessation of breastfeeding at six months [17], their most recent guidelines (2009) support the continuation of breastfeeding up to twelve months, after six months of exclusive breastfeeding [19], along with the introduction of other foods and fluids. Additionally, at this time, breastfeeding should only be stopped if an alternative nutritionally adequate diet can be provided [1]. Review of the international literature highlights difficulties for health care workers conveying best practice recommendations for infant feeding, especially for HIV-positive mothers [22-24]. A health care workers’ lack of up-to-date evidence and informed knowledge subsequently impacts on a mothers’ ability to provide safe infant feeding to their children [25,26], with women often not adhering to best infant feeding practices to reduce HIV transmission [23,27].

This study was designed in 2008, when Papua New Guinea (PNG) was classified as having a generalized HIV epidemic [28]. Since that time estimates have been progressively revised downwards to less than 1% among adults aged 15–49 years [28]. But HIV infection rates in PNG are still among the highest in the Asia Pacific Region, and unlike most other countries of the region, transmission is predominantly heterosexual. Overall antenatal clinic prevalence in 2010 was 0.5% [29]; HIV prevalence among pregnant women varies across the country with the highest rates in Enga (1.4%), Western Highlands (1.3%) and the National Capital District (1.2%) [29].

Initiated through the National Department of Health in 2004, PMTCT services are now available in all provinces in PNG, however coverage remains low [30]. In PNG only 60% of women attend antenatal clinic [31], therefore many women are not screened for HIV and if positive, prescribed ART (prophylaxis and treatment) for either herself or for preventing mother-to-child transmission (ART prophylaxis). Antenatal clinic provider initiated HIV counseling and testing services currently reach only 24% of pregnant women [32]. In 2009 only 12.2% of the estimated 2,150 HIV-positive pregnant women were recorded as having received ART (prophylaxis and treatment) to reduce the risk of mother-to-child transmission [30].

PNG has a culture of breastfeeding, however it is not one of exclusive breastfeeding. Infants are often mix-fed with foods and fluid other than breast milk from as early as a few weeks old [33,34]. This practice poses significant risks to the infant, especially HIV exposed infants. Improving rates of exclusive breastfeeding to six months is recognized in PNG as crucial to achieving better nutrition throughout childhood [35] and to improved infant outcome regardless of HIV status.

Reflecting the WHO, UNICEF and UNAIDS joint statement (1997) and with evidence from other resource poor settings [12,36] the Government of Papua New Guinea developed the first country-specific guidelines relating to HIV and infant feeding in early 2009. Prior to this PNG followed the 2001 WHO guidelines [17]. These guidelines complement national guidelines relating to infant feeding (2009) [35], which seek to protect, promote, and support breastfeeding for all mothers regardless of their HIV status. The guidelines explicitly recommend exclusive breastfeeding for infants of HIV-infected mothers for the first six months of life; replacement feeding is only supported if it is acceptable, feasible, affordable, sustainable and safe [37]. Throughout this six month period of exclusive breastfeeding the mother must continue to receive ART [35]. Reiterated within these guidelines is the Baby Feeding Supplies (Control) Act 1977 (amended 1984), an act to limit the availability of feeding bottles and to ensure that those who use them know how to use them safely [38]. Specifically the aim was to prevent the spread of bottle feeding into rural areas [38]. While this legislation is still in place, feeding bottles, teats, and other feeding aids are freely available in pharmacies throughout PNG without such a prescription.

In line with the national guidelines for the protection, promotion, and support of breastfeeding [37] and the Papua New Guinea Child Health Plan [39], infant feeding guidelines are outlined in the Guidelines on HIV care and treatment in Papua New Guinea [40]. The current infant feeding options available in PNG described within these national guidelines are clearly outlined (Table 1). Although the guidelines unmistakably state that breastfeeding is likely to produce the best health outcome for infants, in relation to both morbidity and mortality, the importance of individual circumstances and a mother’s choice underpins both the information provided and how that information is discussed. Reflecting the WHO revision, the policy in PNG now recommended a significant shift in mother’s feeding practices. HIV-exposed infants were no longer meant to be abruptly weaned from the breast at four to six months but rather continue exclusive breastfeeding to six months and then continue breastfeeding at the same time as the introduction of other food and fluids.

**Table 1:** **Summary of infant feeding options in Papua New Guinea **[40]

**Options**	**Description**
Option 1: Exclusive and continued breastfeeding	Exclusive breastfeeding - nothing other than breast milk is given to the baby for the first six months. Continued Breastfeeding means the continuation of breastfeeding after the introduction of other fluids and food at six months. In addition to the promotion of breastfeeding mothers are taught proper breast attachment to reduce the risk of subsequent breast problems (e.g. mastitis, breast abscess, and engorgement). Maternal combined ARV therapy should be continued to help reduce the risk of transmission of HIV through the breast milk.
Option 2: Express and heat-treat breast milk	While heat treating breast milk offers an ideal nutrition for the baby and has some protection against infections and a low risk of HIV transmission, in nearly all circumstances in PNG this option will be impractical.
Option 3: Breastfeeding by another woman	Also referred to as ‘Wet Nursing’ the woman chosen to breastfeed the infant should be counseled, tested and shown to be HIV-negative. A wet nurse should have access to breastfeeding support and assistance to establish effective breastfeeding.
Option 4: Artificial or replacement feeding from birth	Except in rare situations artificial and replacement feeding is not an option in the PNG context due to the risks involved with replacement feeding in resource limited settings resulting in significantly higher infant mortality.

The aim of this paper is to explore health care workers’ knowledge regarding infant feeding options in PNG. Specifically it aims to examine the extent to which their knowledge reflects the current recommendations outlined in the Papua New Guinea National HIV Care and Treatment Guidelines on HIV and infant feeding (2009).

## Methods

As part of a study investigating women’s and men’s experiences of the Prevention of Mother to Child Transmission (PMTCT) program in PNG, key informants were interviewed in two sites in PNG, Mount Hagen in the Western Highlands Province and Port Moresby in the National Capital District. These sites were identified by a Research Advisory Group meeting in 2009 because they each have high HIV prevalence with well-established PMTCT, HIV and maternal and child health (MCH) services. Antenatal clinic HIV prevalence rates in these sites in 2010 were 1.3% (Western Highlands) and 1.2% (National Capital District) [29]. Informants were purposively selected based upon their involvement in PMTCT and MCH and were drawn from government, non-government and faith based services, reflecting the diversity of such services in PNG.

An interview guide for key informant interviews was developed in parallel with interview guides for women and men engaged in PMTCT programs. The guides were finalized by the research team, piloted and adapted as necessary. The guides were designed to triangulate data from services users and to elicit further details on providing PMTCT services in PNG. All interviews were conducted in a private location in the workplace and lasted between 45 and 90 minutes.

In depth interviews with twenty-eight key informants across the two sites took place between March and November 2011. All but two interviews were conducted in English and transcribed and cross-checked by the lead author. The two interviews conducted in Tok-pisin (a lingua franca of Papua New Guinea) were transcribed verbatim and translated into English.

Key informants included twenty-four health care workers: sixteen nurses/paediatric nurses or midwives and eight senior medical officers working in the field of HIV, obstetrics or pediatrics. Of these, twenty-two were in clinical practice at the time of the interview. The two not in clinical practice were in senior management and advisory positions within the field of MCH/HIV. Seven of the twenty four health care worker informants held positions specifically relating to PMTCT. The remaining four informants included a social worker, a provincial AIDS council coordinator and two People Living with HIV (PLHIV) patient experts.

More than half of the key informants in clinical practice reported having attended a PMTCT training program. The majority of these were nurses or midwives of whom four were PMTCT program managers or coordinators and four were sisters in charge of their respective clinical areas (well baby clinic, labour ward, and antenatal clinic). The remaining informants included senior medical officers, a voluntary counseling and HIV testing (VCT) counselor, a nurse working in a well-baby clinic and a midwife from antenatal clinic.

A code book was developed to thematically analyse the data using NVivo v.9, a qualitative software management program. Codes included those that were inductive that emerged from the data and deductive codes which were used to design the interview guide and drew upon theoretical and empirical findings from other studies. Coding was undertaken by the lead author and cross-checked by the second. To ensure confidentiality, all informants were assigned a pseudonym. For those in clinical practice only their clinical setting is mentioned in this paper, for example well baby clinic.

Ethical approval was gained from the Papua New Guinea Institute of Medical Research Internal Review Board (PNGIMR IRB: 0914), the Medical Research Advisory Committee (MRAC9.25) and the Research Advisory Committee of the National AIDS Council in Papua New Guinea (RES09 010). In Australia ethics approval was gained through the accredited Human Research Ethics committee of University of New South Wales (09208). Written consent to participate in the study was gained from all informants. This research was funded through the Australian Development Research Award Scheme.

## Results

Our findings are presented as they relate to the feeding options described in the current 2009 national guidelines (Table 1).

### Exclusive and continued breastfeeding

Of the feeding options outlined in the Papua New Guinea HIV and infant feeding guidelines, most informants discussed option (1): *Exclusive and continued breastfeeding.* Exclusive breastfeeding was mentioned by almost all informants, the majority of whom reflected the most up-to-date national guidelines for exclusive breastfeeding to six months. Others who discussed this feeding option did not specify how long an HIV-positive mother should exclusively breastfeed.

“I usually tell the mothers that after they have delivered don’t give any cold water. Don’t give even any juice or anything [it] is forbidden to positive mothers. I said [it’s] very forbidden . . . Give only this breast milk until six months . . . When the child reaches four months and five months it will want to eat and will suck its hands and such, don’t give anything. . . only give breast milk for some time until it reaches six months”. (Volunteer Ato, PLHIV patient expert, antenatal clinic)

The continuation of breastfeeding after six months was the area in which key informants appeared to have the least accurate knowledge of current guidelines. While some key informants mentioned that breastfeeding should not continue beyond six months in HIV-exposed infants, others did not mention whether or not breastfeeding should continue after that time. Of those who mentioned exclusive breastfeeding to six months, half mentioned that breastfeeding should continue after the introduction of foods and other fluids at six months. Of the informants reflecting the national breastfeeding guidelines, the majority were senior members of health staff reflecting knowledge gained through access to timely training and policy changes. Other informants who mentioned the continuation of breastfeeding after six months were a well-baby clinic sister and a PLHIV patient expert volunteer working in an antenatal clinic.

For those informants who had attended a PMTCT training program knowledge relating to the recommendation of continuing with breastfeeding after the introduction of food at six months varied, yet continuation after six months is critical to reducing overall infant mortality and morbidity, irrespective of HIV. The majority of these informants knew that infants should exclusively breastfeed for six months. A minority of these highly trained and experienced health care professionals were aware of the importance of the continuation of breastfeeding after the introduction of other food and fluids. A few informants stated that breastfeeding should not continue at this time - two of whom did not know that infants should exclusively breastfeed for six months. The remaining informants did not specify whether breastfeeding should continue or not after the introduction of food and other fluids.

A minority of the informants who discussed exclusive breastfeeding mentioned the importance of the mother continuing with ART while breastfeeding, in line with the current Papua New Guinea HIV and infant feeding guidelines. One informant described how she encouraged mothers to exclusively breastfeed at least until the infant has been tested for HIV.

“. . . we said if you are adherent we’d like you to breastfeed up to 6 months, the Health Department policy is 6 months. The babies are tested at 6 weeks, if you are working and you have money and all that sort of thing, fridge, everything you can afford if you want to put your baby on the formula it’s up to you and your husband. But you have to seriously consider if you can maintain that formula feeding otherwise breast is best . . . you just have to stick to breast but some ask if they can [artificial feed] right at birth and I tell them it’s not wise because what if the babies positive at 6 weeks then all that money on starting the baby on formula is wasted. It’s best to be adherent to your ART and test at 6 weeks”. (Sr Nepina, PMTCT coordinator)

Only a minority of informants did not discuss exclusive and continued breastfeeding at all. The fact that they did not specifically discuss this feeding option is likely to be a reflection of their role and capacity within their work place. The doctors included within this minority group were in fact involved to differing degrees in the formation of the country’s HIV feeding guidelines, but did not work in the PMTCT clinic and were therefore not in a position to provide specific education and information to mothers relating to infant feeding practices.

### Express and heat treat breast milk

While listed as the preferred second option for HIV infant feeding in Papua New Guinea national guidelines only two informants mentioned this option. Both informants expressed a feeling that this option would be too difficult for the majority of mothers in terms of financial implications and time as well as issues relating to hygiene and women would more likely opt to breastfeed.

“You could teach them things like boil the breast milk and you know the other things like the WHO says you can do and reduce the risk but a set up like this [women living in settlements] does not have that kind of thing, who’s going to do it, who’s going to you know have time to do all those things . . .” (Dr Leota, Paediatrician)

### Breastfeeding by another woman

Although mentioned as the third most suitable feeding option for mothers in PNG, only one participant discussed this feeding option. This key informant clearly listed all the feeding options as described in the 2009 HIV infant feeding guidelines.

“. . . the other one [option] is heat and treat . . . we just provide all the information . . . they choose (an option) . . . but they haven’t chosen heat and treat or wet nursing. In most of my counselling that has been done I’ve realized that the two other options are not taken on-board” (Sr Zamila, ANC & breastfeeding advocate).

### Artificial or replacement feeding

Highlighted within the national HIV feeding guidelines as the last option to be used in rare situations, artificial or replacement feeding was the second most commonly mentioned feeding method among informants. Of the informants who discussed replacement or artificial feeding, almost all mentioned this option within the context of mothers specifically requesting information or support relating to this option. They recognised that in many situations it would not be an easy option for mothers to choose, and four mentioned how they explained to mothers about the increased risk of their infant dying from diarrhoeal illness or malnutrition rather than from HIV if they artificially feed their infants.

“. . . we have some who have like have lost their first or second child and this is like their third child and they are not willing to even risk that little percent that will come through the breast milk. So they opt to just bottle feed straight away . . . they think any kind of breastfeeding will bring the virus over. Even if its exclusive breastfeeding they don’t have enough knowledge to say that [make that choice]. . . But now, if the counselling has been given very well, I have spoken to a few mothers . . . who have been counselled through Susu Mama and counselled through the other clinic and they’ve been told that you have to exclusively breastfeed for 6 months and they have”. (Dr. Min, paediatrician)

“. . . because your baby won’t die of HIV, it will die from bottle feeding if you are not careful.” (Sr Naomi, well baby clinic)

### Infant feeding options and the idea of informed choice

A number of the informants who discussed feeding options specifically discussed the importance of providing mothers with necessary information relating to feeding options, enabling them to make an informed choice based on their individual circumstances.

“. . . we don’t choose for them [the mothers] we just tell them the advantages and the disadvantages of it [the option they have chosen]. So they choose for themselves, we do not choose for them . . . then we ask them [if they choose formula feeding], whether the parents are working, are you able to buy milk for one year? And then sometimes we tell them go and sit down with your husband and talk it over again and see which option is best for you”. (Sr. Naomi, Well baby clinic)

A small number of these informants described the importance of informed choice within the context of Papua New Guinean culture, particularly the culture of blame and retribution. Specifically they discussed the importance of choice in relation to feeding options with mothers choosing the most suitable option themselves. It was felt that this is necessary in order to protect the health care worker from any repercussions if an infant was later diagnosed as HIV-positive.

“. . . we give them options, we just don’t want to tell them to continue on breastfeeding and all that [because] later [if] they find that they have problem with the baby, we don’t want them to blame us so we give them option we just say choose, what do you want to choose to feed them . . .” (Sr Kaira, antenatal clinic).

The majority of informants who discussed feeding options described that while the feeding options of exclusive breastfeeding and artificial feeding are discussed with mothers, breastfeeding was ultimately encouraged or emphasized. They went onto explain that breastfeeding is the most suitable option within the context of PNG because of the high financial burden of replacement feeding (approximately USD$40 for one tin of formula milk) in a country with around 80% unemployment, as well as the challenges associated with ensuring adequate hygiene standards particularly with those living in rural areas and settlements.

“Some they can afford to buy this bottle and give them but these things are very expensive and then it is not safe for the baby too so we are encouraging them to breastfeed”. (Sr. Karim, Labour ward)

“[After] the babies are born we normally follow the protocol and see it’s in place. . . the mother is on treatment and we normally encourage exclusive breastfeeding. Because of the cost and also . . . the reason of the hygiene . . .” (Dr Nais, Obstetrician)

## Discussion

Since the introduction of the PMTCT program in PNG in 2004 health care workers have been exposed to changing advice relating to infant feeding. Specifically, the shift in thinking from abrupt cessation of breastfeeding HIV-exposed infants at four to six months, as advised by WHO in 2001, to the continuation of breastfeeding after the introduction of food and other fluids at six months, as advised in 2009 and in line with recommendations from the WHO.

As part of a wider study we explored health care workers knowledge relating to HIV and infant feeding. These findings highlight that while the majority of informants were familiar with the national policy of exclusive breastfeeding, this was less so in relation to the continuation of breastfeeding after the first six months, by far the most important change. Despite the recommendation from the National Department of Health for dissemination of the 2009 infant feeding guidelines, responses from our informants indicate that more than two years after the policy was released the updated information has not been adequately disseminated. The importance of clarity regarding infant feeding recommendations for HIV-positive mothers and the need to integrate new guidelines to all relevant levels of the health system through a systematic approach is highlighted in other parts of the globe [23-25,27].

While many informants in our study discussed the importance of providing the necessary information enabling mothers to make an informed choice about their preferred feeding practice, the idea of choice is somewhat nebulous. Although the health care workers say that they give choice, the guidelines actually limit choice. For example, it states in the guidelines that artificial or replacement feeding “is not an option in the PNG context” [35]. Furthermore because health care workers were providing out dated information on best practices for safe feeding, as suggested by their narratives, women’s ability to make an informed choice was compromised. Moreover, as a result of the power dynamics between patients and healthcare workers who are positioned as experts transferring knowledge about infant feeding is never an objective process, as implied by the informants. A similar concern regarding up-to-date health care worker knowledge of safe infant feeding practices is well documented in Southern Africa [23,24,26]. The risks associated with dissemination of out dated feeding advice in terms of infant health are not only in terms of HIV but also diarrhoeal diseases, malnutrition and mortality [13]. As long as health care workers continue to disseminate out dated information, women will continue to practice unsafe infant feeding. It is incumbent upon PMTCT service providers to ensure they convey the most up to date information in order for women to protect their infants [23,25].

None of the health care workers in this study reported that women expressed a lack of trust about their expertise in PMTCT as a result of discussing feeding options, rather than giving directives. This is in contrast to international research, which has shown that health care workers report women doubting their expertise and thus not trusting them because they offer options and not directives [22].

Our study shows that although four feeding options are listed in the guidelines only two were discussed at any length by our informants. Rather than reflecting poor knowledge, this study highlights what is relevant to PNG and its women. Similarly, international literature on HIV and infant feeding reinforce this emphasis [27].

## Conclusions

Providing advice on optimal infant feeding in resource poor settings is problematic, especially in relation to HIV transmission. Findings from our study reflect those found elsewhere in identifying that key health care workers are not aware of up to date information relating to infant feeding, especially within the context of HIV. This lack of up-to-date information and knowledge leads to inaccurate information being relayed to women, especially in regard to the importance of exclusive breastfeeding for six months with continuation of breastfeeding at this time with the introduction of other foods and fluids. In PNG there is a need to prioritise the dissemination of the 2009 revised infant feeding guidelines thus ensuring those involved in PMTCT services are able to provide optimal advice to all mothers.

## Abbreviations

AFASS: Acceptable feasible, affordable, sustainable and safe; ANC: Antenatal care; ART: Antiretroviral therapy; HIV: Human immunodeficiency virus; MCH: Maternal & child health; NDoH: National department of health; PNG: Papua New Guinea; PNGIMR: Papua New Guinea institute of medical research; PMTCT: Prevention of mother to child transmission; UNAIDS: Joint United Nations program on HIV/AIDS; UNICEF: United Nations international children’s fund; VCT: Voluntary counseling and testing; WHO: World health organization.

## Competing interests

The authors declare that they have no competing interests.

## Authors’ contributions

LV assisted in the designing of the interview guides, was responsible for undertaking most of the interviews, coded and analysed all of the key informant data and led the writing of the manuscript. AK, HW and JK were responsible for the design of the study. AK was responsible for overall running of the project, designing of interview guides, assisted with data analysis and contributed to the writing of the manuscript. HW, GM & JK reviewed the manuscript. MK coordinated all field work for this study and reviewed this manuscript. VF & RN assisted with overall field work, transcribing and translation of interviews and reviewed the manuscript. All authors have read and approved the final manuscript.
